# Xiao-Yao-San protects against anti-tuberculosis drug-induced liver injury by regulating Grsf1 in the mitochondrial oxidative stress pathway

**DOI:** 10.3389/fphar.2022.948128

**Published:** 2022-09-01

**Authors:** Zijun Bai, Weiwei Tao, Yiqun Zhou, Yi Cao, Shun Yu, Zheng Shi

**Affiliations:** ^1^ School of Chinese Medicine, School of Integrated Chinese and Western Medicine Nanjing University of Chinese Medicine, Nanjing, Jiangsu, China; ^2^ Ningxia Key Laboratory of Cerebrocranial Disease, Incubation Base of National Key Laboratory, College of Pharmacy, Ningxia Medical University, Yinchuan, Ningxia, China; ^3^ Department of Infectious Disease, Suzhou Integrated Chinese and Western Medicine Hospital, Suzhou, Jiangsu, China; ^4^ Institute of Literature in Chinese Medicine, Nanjing University of Chinese Medicine, Nanjing, Jiangsu, China

**Keywords:** Xiao-Yao-San, ferroptosis, Grsf1 shRNA, DILI, RUCAM, anti-tuberculosis drugs, mitochondria synthesis

## Abstract

**Background:** Xiao-Yao-San (XYS) is a traditional Chinese prescription that regulates gastrointestinal function, improves mental and psychological abnormalities, and enhances liver function. However, the underlying mechanism of XYS for relieving anti-tuberculosis (AT) drug-induced liver injury is not clear.

**Objective:** The current study examined whether XYS alleviated the symptoms of AT drug-induced liver injury in mice via the mitochondrial oxidative stress pathway.

**Methods:** BALB/c male mice were randomly divided into four groups of 12 animals, including a control group, a model group, a 0.32 g/kg XYS group, and a 0.64 g/kg XYS group. The effect of XYS on the degree of liver injury was observed using haematoxylin and eosin staining (HE) and oil red O staining of pathological sections, biochemical parameters, and reactive oxygen species (ROS) levels. The protein expression of mitochondrial synthesis-related proteins and ferroptosis-related proteins was examined using Western blotting.

**Results:** XYS improved the pathological changes in liver tissue and reduced the level of oxidative stress in liver-injured mice. XYS increased the expression of mitochondrial synthesis-related proteins and reversed the expression of ferroptosis-related proteins. Knockdown of G-rich RNA sequence binding factor 1 (Grsf1) expression with Grsf1 shRNA blocked the protective effects of XYS in liver injury.

**Conclusion:** Our findings suggest that XYS alleviates AT drug-induced liver injury by mediating Grsf1 in the mitochondrial oxidative stress pathway.

## Introduction

Tuberculosis (TB) continues to affect people’s health as a global public health problem (Mahase, 2019). China is the third largest source of new TB cases globally, and it accounts for 8.4% of the global total (WHO, 2020). However, most of the first-line drugs used to treat TB have potential hepatotoxic effects (Kesenogile et al., 2021). Drug-induced Liver Injury (DILI) is an injury to the liver or biliary system associated with the ingestion of hepatotoxic drugs, and it is one of the most common causes of acute liver failure (Devarbhavi et al., 2018). A crucial first step in patients with suspected DILI receiving hepatotoxic drugs is to stop taking the medicine in question (Andrade et al., 2019). Anti-tuberculosis drug-induced hepatotoxicity (ATDH) is the most common adverse effect of anti-TB treatment, and it makes it difficult for patients to adhere to the entire course of anti-tuberculous chemotherapy (AC) (Cavaco et al., 2022). Drugs available for the treatment of liver injury, such as silymarin, have limited clinical use due to their poor pharmacological effects (Flora et al., 1998). N-acetylcysteine and ursodeoxycholic acid have also been used to treat liver injury, but there is little evidence to support this treatment (Andrade et al., 2019). Therefore, the development of new drugs for the treatment of liver injury has become a global research hotspot.

Prescription is the primary method of treatment in traditional Chinese medicine in clinical practice (Alcorn and Ouyang, 2012). Xiao-Yao-San (XYS) originated from *Taiping Huimin Heji Jufang* (Song Dynasty, 960-1127 CE). XYS has a long history of clinical use, and it regulates gastrointestinal function, improves mental and psychological abnormalities, and enhances liver function (Chien et al., 2014; Liu et al., 2021; Lin et al., 2022). Since the 1990s, several randomised controlled trials (RCTs) were performed and confirmed the remarkable therapeutic pharmacological effects of XYS in the treatment of ATDH (Hu, 2008). XYS contributes to adherence to the entire AC course, which is of great significance to the success of anti-TB treatment. Previous studies focused on revealing the mechanisms underlying the antidepressant effects of XYS (Chen et al., 2020; Zeng et al., 2022), but there is a lack of research on the mechanisms by which XYS attenuates liver damage.

Grsf1 is an RNA-binding protein that is widely found in body tissues and belongs to the F/H family of heterogeneous nuclear ribonucleic acid proteins (Ufer, 2012). Grsf1 is an essential protein for oxidative phosphorylation in mitochondria, and it influences mitochondrial function by participating in RNA processing, translocation, and translation to play an essential role in maintaining mitochondrial homeostasis, which is the main site of oxidation in organisms (Bayona-Bafaluy et al., 2011; Jourdain et al., 2013).

We investigated the application of XYS to treat liver injury using a BALB/c male mouse model of liver injury. Isoniazid is a hepatic enzyme inhibitor, and rifampicin is a hepatic enzyme inducer. Both of these agents are anti-tuberculosis (AT) drugs. Isoniazid and rifampicin induce DILI in clinical practice, as assessed by the Roussel Uclaf Causality Assessment Method (RUCAM), which was updated in 2016 (Cavaco et al., 2022). The current study constructed a mouse model of AT drug-induced liver injury using 100 mg/kg rifampicin combined with 50 mg/kg isoniazid (Chowdhury et al., 2006). Based on the pharmacological effects assessment, we investigated the protective effect of two concentrations of XYS on liver-injured mice. Pathway-based investigations interfered with the Grsf1 gene and examined the protective effect of XYS in mice with liver injury in the presence of Grsf1 inhibition.

## Methods and materials

### Antibodies and reagents

Anti-peroxisome proliferator-activated receptor *γ* coactivator 1 alpha (PCG-1α) antibody (ab191838), anti-nuclear respiratory factor 1 (NRF1) antibody (ab175932), anti-mitochondrial transcription factor A (TFAM) antibody (ab176558), anti-ferritin heavy chain 1 (FTH1) antibody (ab65080), anti-transferrin receptor (ab269513), anti-glutathione peroxidase 4 antibody (ab125066) and anti-Grsf1 (ab205531) were purchased from Abcam. Rifampicin (R817237), isoniazid (I811711), and silybin (S817883) were purchased from Macklin Inc.

### Preparation and assessment of Xiao-Yao-San

XYS is composed of eight botanical drugs. All drugs were purchased from Nantong Sanyue Chinese Medicine Decoction Pieces Company (Jiangsu, China) and authenticated by Professor Jianwei Chen, Nanjing University of Chinese Medicine. XYS was prepared following the Chinese Pharmacopeia 2020 Edition (0189). Fifteen grams of *Bupleurum chinense DC.* (Apiaceae, Bupleuri Radix), 15 g of *Angelica sinensis* (Oliv.) Diels (Apiaceae, Angelicae Sinensis Radix), 15 g of *Paeonia lactiflora* Pall. (Paeoniaceae, Paeoniae Radix Alba), 15 g of *Atractylodes macrocephala* Koidz. (Compositae, Atractylodis Macrocephalae Rhizoma), 15 g of *Poria cocos* (Schw.) Wolf (Polyporaceae, Poria), and 15 g of *Glycyrrhiza uralensis* Fisch. (Leguminosea, Glycyrrhizae Radix et Rhizoma) were crushed into a powder, and 1,500 ml (15/1; v/w) of distilled water was added. These botanical drugs were boiled for 20 min, and 5 g of *Zingiber officinale* Roscoe (Zingiberaceae, Zingiberis Rhizoma Recens) and 5 g of *Mentha haplocalyx* Briq. (Lamiaceae, Menthae Haplocalycis Herba) were added*.* Then, all of the botanical drugs were boiled for 10 min. The decoction was passed through five layers of gauze, and the filtrate was concentrated under reduced pressure and made into the extract. The yield of the XYS extract was 16% (extract weight/crude drug weight). The production process of the XYS extract was described in Supplementary Figure S1. The doses of XYS used in animal experiments (0.32 and 0.64 g/kg) are expressed as the weight of XYS extract (g)/body weight (kg).

The main components of XYS are ferulic acid (F8330, Solarbio), paeoniflorin (SP8030, Solarbio), ligustilide (SL8120, Solarbio), liquiritin (SL8210, Solarbio), and glycyrrhizic acid (SG8600, Solarbio). For quality control, the XYS components in this study were measured using high-performance liquid chromatography with diode array detection (HPLC-DAD) (Waters Corporation, United States).

### Animals and experimental protocol

Isoniazide affects the antioxidant enzyme system of the liver, causes depletion of antioxidants in the liver, exceeds the detoxification capacity of hepatocytes, and causes disruption of liver function, which ultimately lead to oxidative liver injury. When isoniazid and rifampicin are combined, rifampicin is acetylated in the liver, which provides more acetyl groups for isoniazid and aggravates hepatocellular damage (Mann et al., 2017). A BALB/c male mouse liver injury model was constructed using 100 mg/kg rifampicin combined with 50 mg/kg isoniazid, because rifampicin and isoniazid lead to liver glutathione depletion and increased lipid peroxidation levels, which result in hepatocyte apoptosis and liver lipid accumulation (Chowdhury et al., 2006). Liver injury model mice were divided into five groups (12 mice per group): control group, model group, 0.32 g/kg XYS group, 0.64 g/kg XYS group, and 100 mg/kg Si (silybin, positive control). Except for the model group, the mice were treated with the appropriate drugs via gavage from the first day of modelling. The liver injury model mice were divided into four groups in the second group of experiments: Control shRNA + Vehicle group, Control shRNA + XYS (0.64 g/kg) group, Grsf1 shRNA + Vehicle group and Grsf1 shRNA + XYS (0.64 g/kg) group. Mouse Grsf1 (Gene ID: NM_001098476) AAV-shRNA-Grsf1 adeno-associated virus was used to interfere with the sequence 5′-GCG​GTA​TGT​GGA​AGT​GTA​TGA-3′ provided by Shanghai Gene Technology Co. The AAV adeno-associated virus was transfected into mice at a dose of 9 × 10^11^ viral genome copies/mouse, and the virus was injected via the tail vein. Moulding and drug administration were performed 2 weeks after transfection. Mice were administered 100 mg/kg rifampicin combined with 50 mg/kg isoniazid on days 1–3 to produce a liver injury model. On day 4, blood was taken from the abdominal aorta of mice, and kidney tissue was obtained after euthanasia. Vehicle mice received equal amounts of saline via gavage. The procedure was performed in accordance with the Committee on the Ethics of Animal Experiments of Nanjing University of Chinese Medicine (202204A001).

### Haematoxylin and eosin staining

Haematoxylin is a basic dye that stains cell nuclei bluish-purple colour, and eosin is an acidic dye that stains the cytoplasm of most cells red. The tissue was fixed in a 10% formalin solution, dehydrated, cleared in xylene, and paraffin embedded. Sections were cut into 5–8-µm thick sections, dewaxed and stained with haematoxylin and eosin, dehydrated, cleared, resin sealed, and placed under a microscope for observation.

### Detection of biochemical indicators

The serum levels of interleukin (IL)-1β, IL-6, and tumour necrosis factor (TNF)-α were determined using an IL-1β mouse ELISA kit (ZC-36404, ZCIBIO Technology Co., Ltd.), IL-6 mouse ELISA kit (ZC-36391, ZCIBIO Technology Co., Ltd.) and a TNF-α mouse ELISA kit (ZC-37624, ZCIBIO Technology Co., Ltd.). The alanine aminotransferase (ALT, C009-2-1) and aspartate aminotransferase (AST, C010-2-1) assay kits from Nanjing Jiancheng Bioengineering Institute were used to measure liver function and concrete operation according to the manufacturer’s instructions. Superoxide dismutase (SOD), malondialdehyde (MDA), and glutathione (GSH) were measured by reference to SOD (A001-3-2, Jiancheng), MDA (A003-1-2, Jiancheng), and GSH (A005-1-2, Jiancheng) according to the manufacturer’s instructions. Pretreated cells or tissue supernatant were mixed with the reagents in the commercial kit according to the instructions, and placed under a spectrophotometer to read the absorbance value. The corresponding value for each sample was calculated from the standard curve. Total iron levels in the different groups were analysed using an iron assay kit (ab83366, Abcam, United Kingdom). Five microlitres of iron reducing agent was added to 50 μl of pretreated tissue or cell supernatant for the assay, and 100 μl of iron probe solution was added to the samples and incubated in the dark at 25°C for 60 min. The samples were placed under a spectrophotometer to read the absorbance value, and the corresponding value for each sample was calculated from the standard curve.

### Oil red O staining

Oil Red O is a strong lipid solvent and lipid stain that specifically stains neutral triglycerides, lipids, and lipoproteins in tissues and cells. Sections pretreated with 60% isopropanol were placed in oil red O staining solution for 15 min, protected from light, incubated in 60% isopropanol for 1 min, washed and placed in haematoxylin staining solution for 5 min. After rapid fractionation in 1% hydrochloric acid alcohol for 2 s, the sections were rinsed slowly in running water for 2 h, dried, and sealed. The lipid droplet area ratio in oil red O staining was analysed using Image-Pro Plus software.

### Measurement of reactive oxygen species

The level of intracellular ROS was measured using the ROS fluorescent probe 2,7-dichloro-dihydro-fluorescein diacetate (DCFH-DA, No. S0033M, Beyotime). Liver tissues were made into a cell suspension and incubated with DCFH-DA for 30 min. The fluorescence intensity of 2′, 7′-dichlorofluorescein (DCF) was detected using fluorescence microscopy and photographed. The green fluorescence intensity was analysed using ImageJ software, which indicated the relative amount of ROS in the liver tissue.

### Mitochondrial membrane potential detection

JC-1 (J6004, UE, China) is an ideal fluorescent probe for detecting the mitochondrial membrane potential Δ Ψm. A decrease in mitochondrial membrane potential is a marker of early apoptosis. Therefore, the transition of JC-1 from red to green fluorescence is used as an indicator of early apoptotic cell death. After incubation at 37°C for 15 min, the supernatant was removed by centrifugation, and the procedure was repeated, followed by re-washing in PBS buffer and assay on the machine.

### Western blot analysis

Liver tissues were collected and stored at −80°C for protein blot analysis. Tissues were homogenised with radioimmunoprecipitation assay buffer and centrifuged. The supernatant was collected, and the total protein concentration was detected using a BCA analysis kit (P0018FS, Beyotime). Equal amounts of proteins were separated using 10% sodium dodecyl sulphate polyacrylamide gel electrophoresis and electroblotted onto polyvinylidene difluoride membranes. After blocking with PBST containing 5% fat-free milk, the membranes were incubated with primary antibodies overnight at 4°C and incubated with horseradish peroxidase (HRP)-conjugated secondary antibodies for 2 h. The immuneblot bands were visualised using enhanced chemiluminescence (ECL) luminescence reagent (K002, Affinity) and quantified using ImageJ software.

### Immunofluorescent staining

Liver sections were incubated with an anti-GPX4 antibody overnight at 4°C and incubated with HRP-conjugated goat anti-rabbit secondary antibody for 1 h, followed by re-staining with 4′,6-diamidino-2-phenylindole (DAPI).

### Statistical analysis

Statistical analyses were performed using GraphPad Prism software. Data are expressed as the means ± standard deviations (SD). Statistical significance was calculated using Student’s *t* test, one-way analysis of variance (ANOVA) and two-way ANOVA. *p* < 0.05 was considered statistically significant.

## Results

### Chemical profiling for Xiao-Yao-San

**FIGURE 1:**
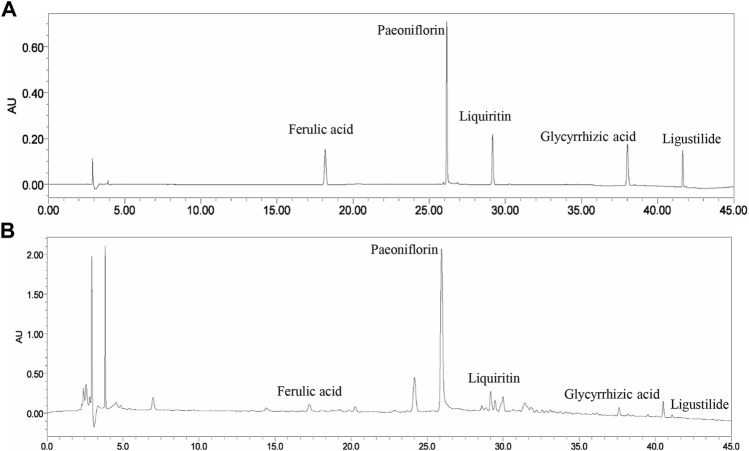
The HPLC fingerprints of XYS and its major components, including ferulic acid, paeoniflorin, liquiritin, glycyrrhizic acid, and ligustilide. **(A)** Standards. **(B)** Samples.

The main components of XYS are ferulic acid, paeoniflorin, liquiritin, glycyrrhizic acid, and ligustilide. As shown in Figures 1A,B, these five components were identified in XYS by comparative analysis of the molecular retention times of the standards and XYS samples: Ferulic acid 0.054 mg/kg, paeoniflorin 1.224 mg/kg, liquiritin 0.062 mg/kg, glycyrrhizic acid 0.925 mg/kg, and ligustilide 0.005 mg/kg.

### Xiao-Yao-San improves liver histopathology and serum biochemical parameters in liver-injured mice

**FIGURE 2:**
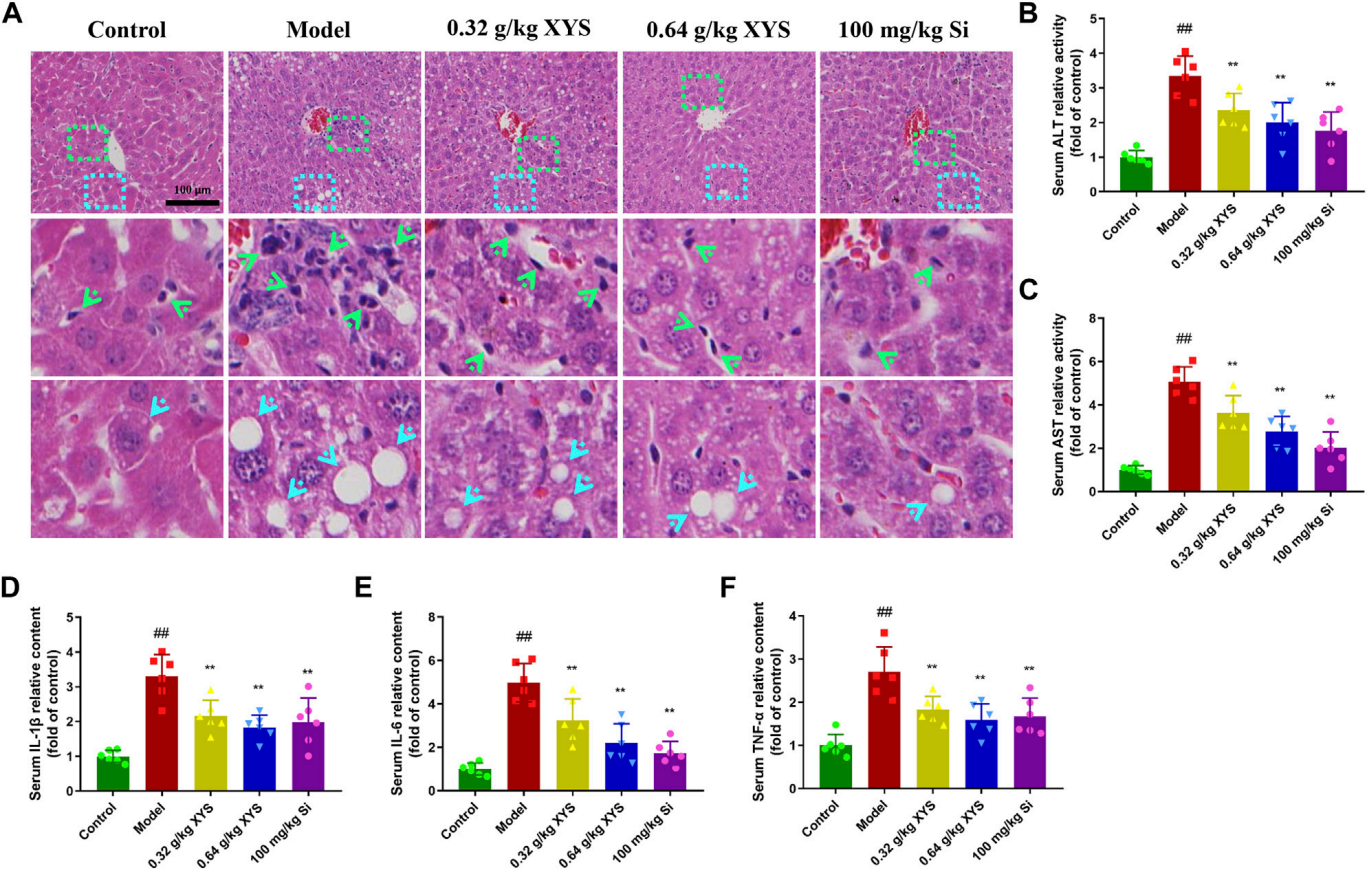
Histopathology of liver and biochemical indicators in serum with the administration of XYS and silybin. Mice were treated with XYS (0.32 or 0.64 g/kg) or silybin (100 mg/kg) via gavage for three consecutive days and sacrificed on the fourth day. **(A)** The histopathology of the mouse liver 24 h after the last administration, and liver sections were stained with haematoxylin and eosin. **(B)** The level of ALT in mouse serum in each group, and each bar represents the mean level of ALT in serum ±SD. **(C)** The level of AST in mouse serum in each group, and each bar represents the mean level of AST in serum ±SD. **(D)** The content of IL-1β in mouse serum in each group, and each bar represents the mean content of IL-1β in serum ±SD. **(E)** The content of IL-6 in mouse serum in each group, and each bar represents the mean content of IL-6 in serum ±SD. **(F)** The content of TNF-α in mouse serum in each group, and each bar represents the mean content of TNF-α in serum ±SD. ^#^ *p* < 0.05 vs. Control group, and ^##^ *p* < 0.01 vs. Control group; ∗*p* < 0.05 vs. Model group and ∗∗*p* < 0.01 vs. Model group.

Co-administration of rifampicin + isoniazid to BALB/c mice for 3 days resulted in the appearance of predominantly vacuolated (blue arrows) and inflammatory cell infiltrates (green arrows) in the liver tissues of the model group, as shown in Figure 2A. Drug-induced liver injury was demonstrated by significant elevations in serum ALT and AST (Figures 2B,C). IL-1β, IL-6, and TNF-α were also significantly elevated in the model group compared to the control group (Figures 2D–F). XYS reduced the levels of ALT, AST, and inflammatory factors and improved the histopathological changes compared to the model group.

### Xiao-Yao-San reduces lipid content in liver tissue of liver-injured mice

**FIGURE 3:**
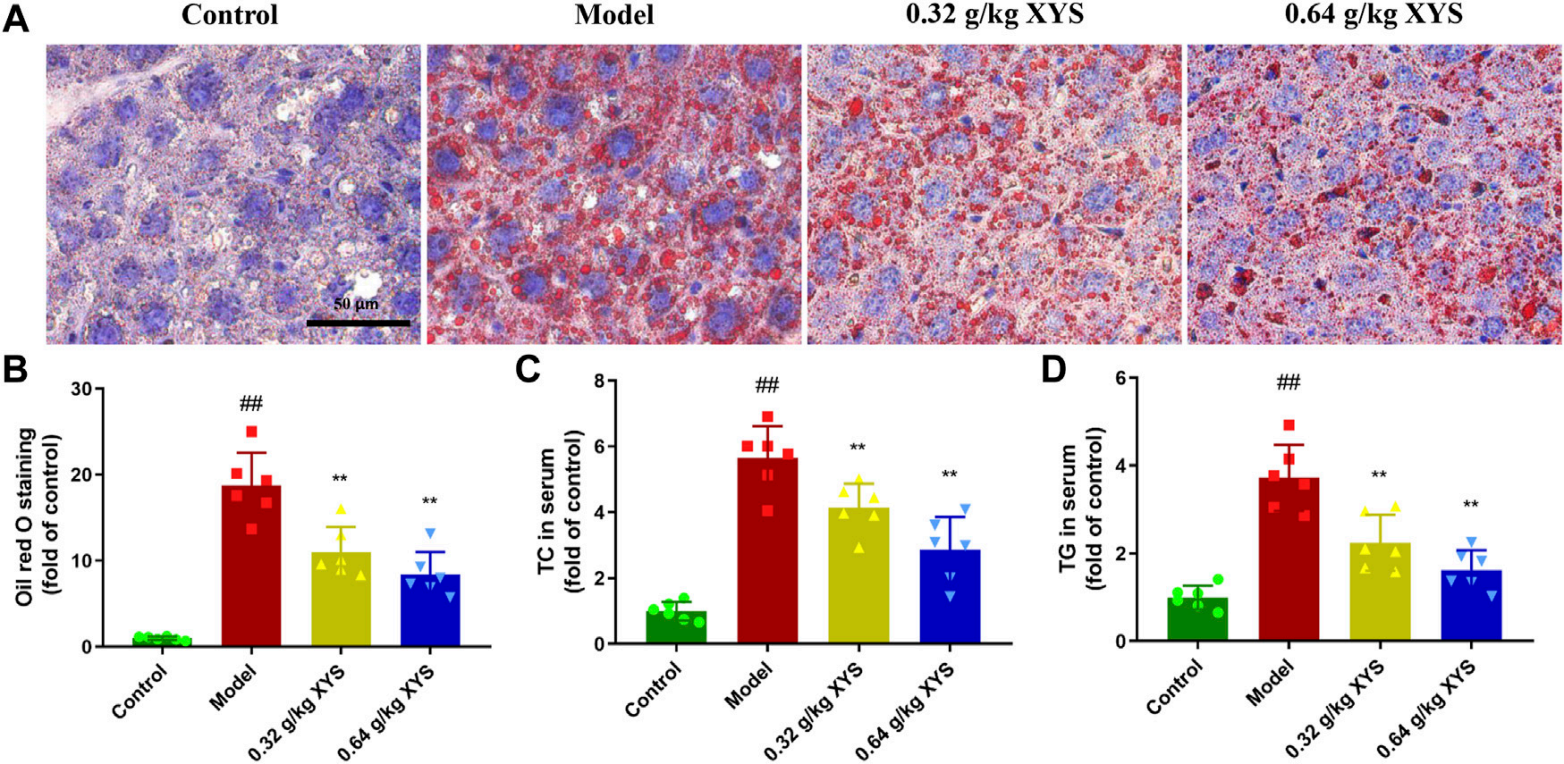
Oil red O staining of liver and lipid biochemical indicators in serum with the administration of XYS. Mice were treated with XYS (0.32 or 0.64 g/kg) *via* gavage for three consecutive days and sacrificed on the fourth day. **(A)** Oil red O staining of mouse liver 24 h after the last administration in each group to detect fat in liver tissues, with red areas representing fat droplets. **(B)** Relative content of oil red O staining in mouse liver tissues in each group, and each bar represents the mean content of oil red in serum ± SD. **(C)** The level of TC in mouse serum in each group, and each bar represents the mean level of TC in serum ± SD. **(D)** The TG content in mouse serum in each group. Each bar represents the mean TG content in the serum ± SD. ^#^ *p* < 0.05 vs. Control group, and ^##^ *p* < 0.01 vs. Control group; ∗*p* < 0.05 vs. Model group and ∗∗*p* < 0.01 vs. Model group.

Oil red O staining of liver tissue showed that sections of liver tissues from the model group showed a large number of stained fat droplets. In contrast, liver sections from the XYS-treated mice had far fewer fat droplets (Figure 3A), and this difference is shown in Figure 3B. As shown in Figures 3C,D, total cholesterol and triglycerides in the livers of the model group of mice were significantly increased compared to the control group, and the use of XYS reduced these two indicators.

### Xiao-Yao-San decreases the levels of oxidative stress and Fe^2+^ in liver tissues of liver-injured mice

**FIGURE 4:**
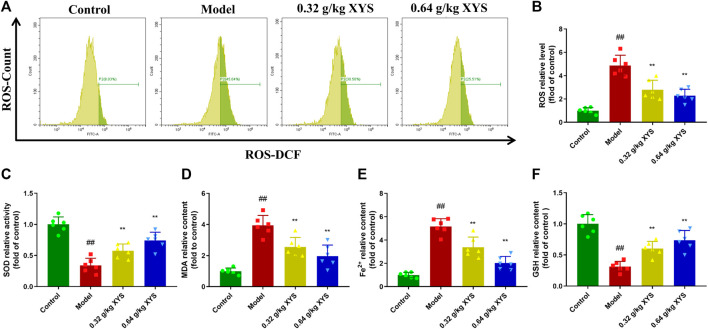
The oxidative stress level and Fe^2+^ production in liver tissues in each group. Mice were treated with XYS (0.32 or 0.64 g/kg) via gavage for three consecutive days and sacrificed on the fourth day. **(A)** The ROS level of cells from liver tissue homogenates in each group was detected using flow cytometry with a DCFH-DA probe. **(B)** The relative level of ROS in each group, and each bar represents the mean level of ROS in liver tissues ±SD. **(C)** The relative level of SOD in each group, and each bar represents the mean level of SOD in liver tissues ±SD. **(D)** The relative level of MDA in each group, and each bar represents the mean level of MDA in liver tissues ±SD. **(E)** The relative content of Fe^2+^ in each group, and each bar represents the mean content of Fe^2+^ in liver tissues ±SD. **(F)** The relative level of GSH in each group, and each bar represents the mean level of GSH in liver tissues ±SD. ^#^ *p* < 0.05 vs. Control group, and ^##^ *p* < 0.01 vs. Control group; ∗*p* < 0.05 vs. Model group and ∗∗*p* < 0.01 vs. Model group.

To determine the oxidative stress in liver tissues, we homogenised the liver tissues to isolate hepatocytes and analysed the DCF-positive cell content using flow cytometry. As shown in Figures 4A,B, ROS levels were substantially elevated in the model group compared to the control group, and XYS significantly reduced ROS levels. The MDA and Fe^2+^ levels of mice in the model group were much higher than the control group. However, SOD activity and GSH levels were reversed. Treatment with XYS reversed this trend (Figures 4C–F).

### Xiao-Yao-San increases mitochondrial membrane potential and the expression of mitochondrial synthesis-related proteins in liver tissues of liver-injured mice

**FIGURE 5:**
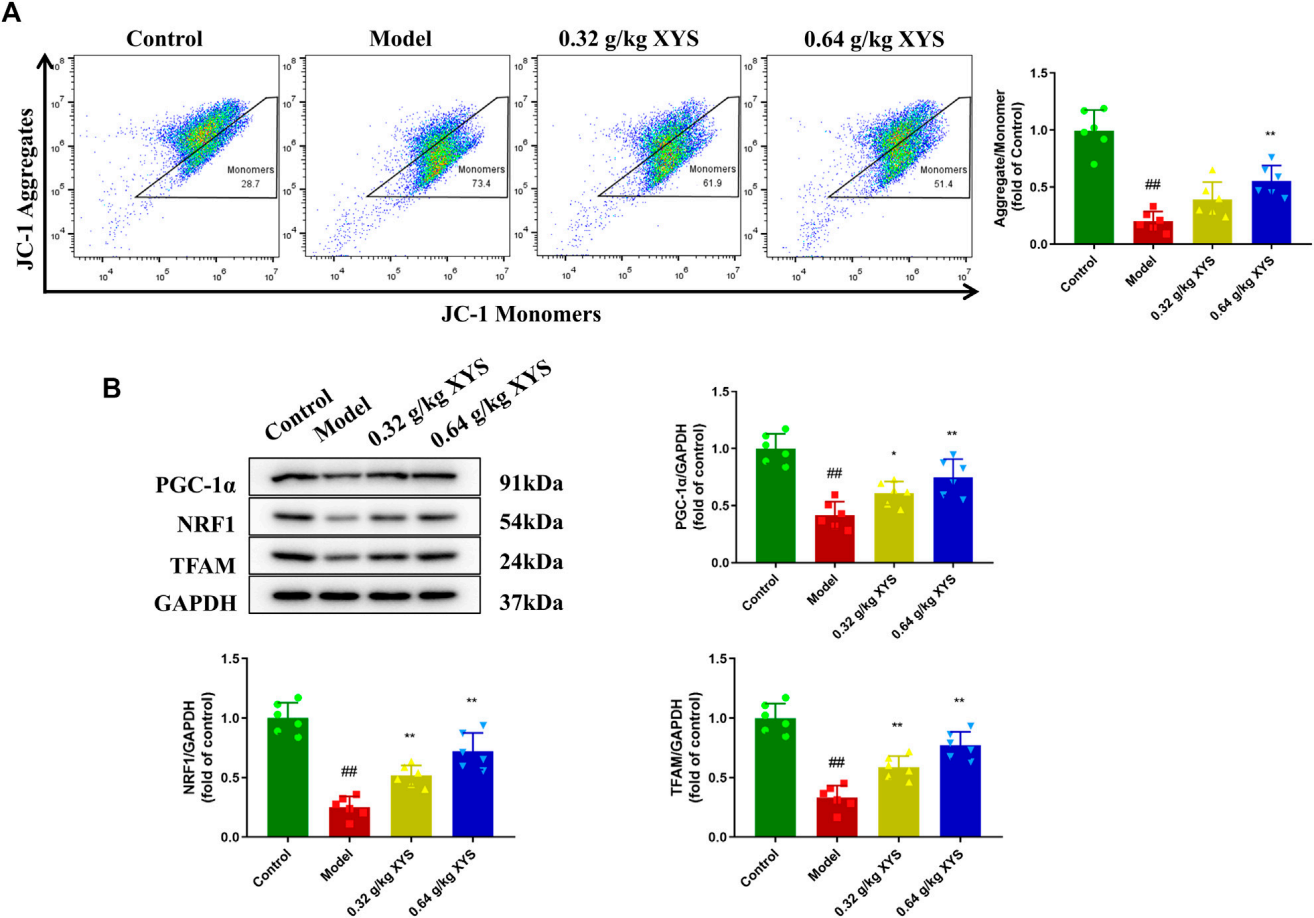
Detection of mitochondrial membrane potential and expression of mitochondrial synthesis-related proteins. Mice were treated with XYS (0.32 or 0.64 g/kg) *via* gavage for three consecutive days and sacrificed on the fourth day. **(A)** The potential of the cell mitochondrial membrane from liver tissue homogenates in each group was detected using flow with a JC-1 probe. **(B)** Representative images of Western blot analysis of PGC-1α, NRF1, and TFAM and their relative quantitative histograms. GAPDH was used as an internal control. ^#^ *p* < 0.05 vs. Control group, and ^##^ *p* < 0.01 vs. Control group; ∗*p* < 0.05 vs. Model group and ∗∗*p* < 0.01 vs. Model group.

Mitochondrial membrane potential was assessed by incubation with JC-1 after cells were isolated from liver tissues of different groups of mice. As shown in Figure 5A, the mitochondrial membrane potential in the model group decreased compared to the control group, which indicated the onset of apoptosis in the model group. The use of XYS increased the mitochondrial membrane potential. We examined the expression of mitochondrial synthesis-related proteins in the context of decreased mitochondrial membrane potential. Reduced PGC-1α, NRF1 and TFAM protein levels were detected in the model group of mice, which correlate with increased oxidative stress. XYS reduced oxidative stress and increased mitochondrial synthesis-related protein expression (Figure 5B).

### Xiao-Yao-San increases the intensity of GPX4 immunofluorescence and alters the expression of ferroptosis-associated proteins in liver tissues of liver-injured mice

**FIGURE 6:**
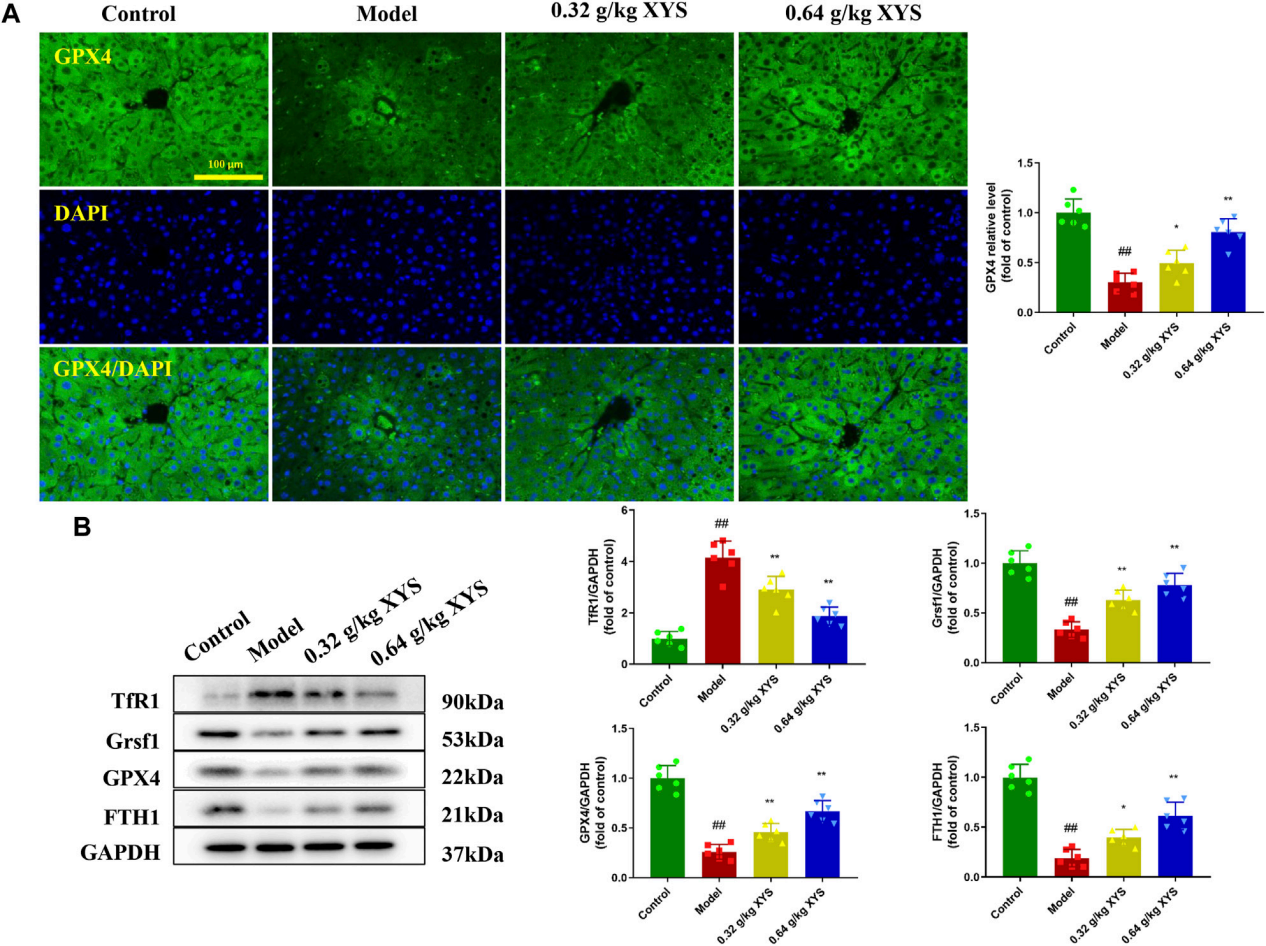
Immunofluorescence of GPX4 and the expression of ferroptosis-associated proteins. Mice were treated with XYS (0.32 or 0.64 g/kg) *via* gavage for three consecutive days and sacrificed on the fourth day. **(A)** Representative immunofluorescent images of GPX4 in liver tissues from each group, and relative quantitative histograms of GPX4, with each bar representing mean level of GPX4 in liver tissues ±SD. **(B)** Representative images of Western blot analyses of TfR1, Grsf1, GPX4, and FTH1 and their relative quantitative histograms. GAPDH was used as an internal control. ^#^ *p* < 0.05 vs. Control group, and ^##^ *p* < 0.01 vs. Control group; ∗*p* < 0.05 vs. Model group and ∗∗*p* < 0.01 vs. Model group.

The GPX4 enzyme is the only intracellular glutathione peroxidase used for liposome peroxide reduction, and depletion of GSH causes GPX4 inactivation. Therefore, we examined the GPX4 content of mouse liver tissue using immunofluorescence. We found that GPX4 fluorescence intensity was reduced in the model group compared to the control group. In contrast, XYS increased the GSH content and the immunofluorescence intensity of GPX4 (Figure 6A). To determine the potential mechanism of XYS against oxidative stress, the expression of the ferroptosis-related proteins TfR1, Grsf1, GPX4, and FTH1 in liver tissue was examined using protein blotting. Pretreatment with XYS significantly upregulated the expression of Grsf1, GPX4 and FTH1 and downregulated the expression of TfR1 compared to the model group (Figure 6B).

### The regulation of mitochondrial synthesis-related proteins and ferroptosis-related proteins by Xiao-Yao-San is abolished when Grsf1 is inhibited

**FIGURE 7:**
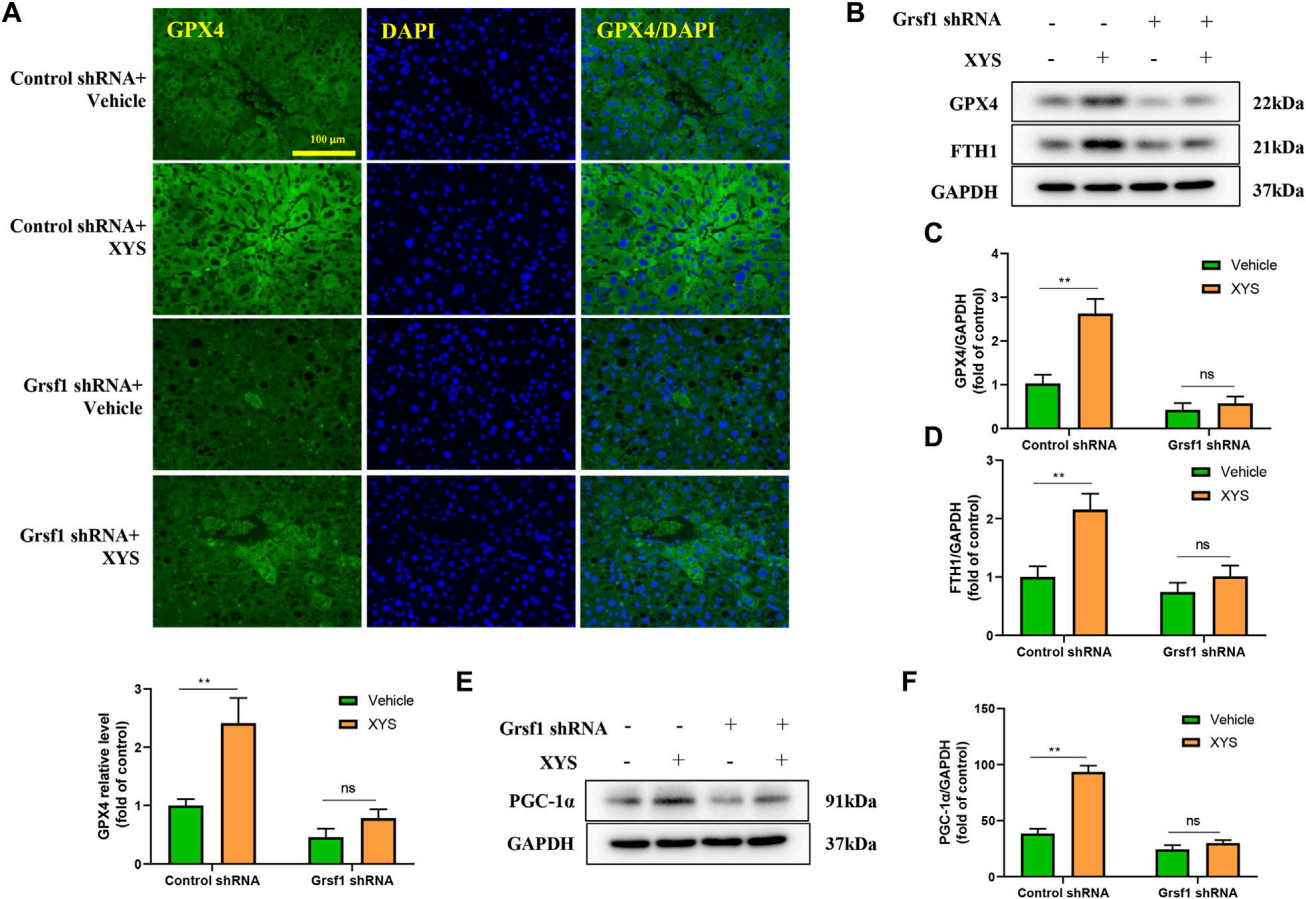
The expression of mitochondrial synthesis-related proteins and ferroptosis-related proteins when Grsf1 was inhibited. Control shRNA mice or Grsf1 shRNA mice were treated with or without 0.64 g/kg XYS *via* gavage for three consecutive days and sacrificed on the fourth day. **(A)** Representative immunofluorescent images of GPX4 in liver tissues from each group and relative quantitative histograms of GPX4. Each bar represents the mean level of GPX4 in liver tissues ±SD. **(B–D)** Representative images of Western blot analysis of GPX4 and FTH1 and their relative quantitative histograms. **(E,F)** Representative images of Western blot analysis of PGC-1α and its relative quantitative histograms. GAPDH was used as the internal control. ∗∗*p* < 0.01 indicates a significant difference, and ns indicates no significance.

To further explore the protective mechanism of XYS against liver injury, we interfered with Grsf1. As shown in Figure 7A, the use of XYS significantly increased the expression of GPX4 in control shRNA mice, but XYS did not significantly affect the expression of GPX4 in Grsf1 shRNA mice. The original promotion of GPX4, FTH1, and PGC-1α protein expression by XYS was inhibited in the presence of Grsf1 disruption (Figures 7B–F).

## Discussion

Isoniazid, rifampicin, pyrazinamide, and ethambutol cause liver damage, which leads to elevated liver transaminases and affects hepatocyte function (Cavaco et al., 2022). The incidence of liver disease increases each year, and the development of drugs to treat drug-induced liver injury has become a hot topic of research worldwide. The experimental results of the present study demonstrated that XYS had a protective effect on drug-induced liver injury and further revealed that the protective effect of XYS against liver injury was associated with mitochondrial synthesis and ferroptosis in hepatocytes.

XYS regulates gastrointestinal function, improves mental and psychological abnormalities, and protects and improves liver function (Chen et al., 2020; Liu et al., 2021; Lin et al., 2022). A retrospective study of 48 patients showed that XYS improved symptoms and reduced relapse rates in patients with functional gastrointestinal disorders (Liu et al., 2021). XYS alone or in combination with anxiolytics improves anxiety-related symptoms and reduces drug-related adverse effects compared to conventional treatment (Lin et al., 2022). A randomised controlled trial of 120 patients with drug-related liver injury showed that XYS treated drug-related liver injury caused by AT drugs and adhered to the standard course of AC (Hu, 2008).

ALT and AST in serum are often used as markers of AT drug-induced liver injury, and their elevated concentrations in serum suggest the severity of liver injury. The present study showed that the levels of ALT and AST in mice of the model group were significantly increased compared to the control group, which demonstrated the successful establishment of an AT drug-induced liver injury model. Cells release TNF-α, IL-6 and other inflammatory factors during liver injury (Olteanu et al., 2012). IL-6 mediates the inflammatory response and tissue repair. TNF-α activates inflammatory factors and is involved in cell proliferation and apoptosis, and its levels directly correlate with the degree of inflammation (Zhang et al., 2018; Bogacz et al., 2020). Therefore, reducing the level of the inflammatory response will help reduce liver injury. IL-10 is one of the main anti-inflammatory factors that can inhibits the effect of the inflammatory mediators IL-6 and TNF-α (Bogacz et al., 2020). We demonstrated that XYS inhibited inflammatory transmitters by lowering the contents of TNF-α and IL-6 in mouse liver tissues and increasing the content of IL-10, which has specific effects on reducing liver injury in mice, similar to the results of a previous study (Chien et al., 2014). XYS improved 23 of 35 potential liver biomarkers disrupted by the induction of chronic extreme mild stress (Chen et al., 2020) and had an anti-fibrosis effect on chronic liver injury induced by dimethylnitrosamine in rats (Chien et al., 2014).

Some of the botanical drugs in XYS and its constituents have hepatoprotective effects. For example, saponin D protected mice from acetaminophen-induced hepatotoxicity by downregulating NF-κB and STAT3-mediated inflammatory signalling pathways (Liu et al., 2014). Extraction of Angelica sinensis polysaccharides (ASP) showed a significant protective effect against CCl4-induced hepatotoxicity, which was due to the antioxidant properties of ASP (Yu et al., 2013). Licochalcone A had a protective effect on lipopolysaccharide/d-galactosamine-induced hepatotoxicity, which may be closely related to Nrf2 activation and autophagy (Lv et al., 2019). The mechanisms of these monomeric actions are all related to oxidative stress pathways. We also explored the relationship between XYS and changes in the levels of oxidative stress.

The antioxidant enzyme systems MDA, SOD, and GSH in the liver are commonly used to evaluate oxidative stress. MDA is an end product of lipid peroxidation, and its content reflects the degree of lipid peroxidation damage in tissues. The level of SOD activity indirectly demonstrates the body’s ability to scavenge oxygen free radicals. The levels of MDA and ROS in the liver tissues of the model mice were significantly increased in the present study, and GSH and SOD were significantly decreased, which indicated that the antioxidant capacity of the liver of the model mice was significantly decreased, and lipid peroxidation was increased. In contrast, XYS significantly reduced MDA and ROS levels and increased GSH and SOD levels. GSH is an essential antioxidant and free radical scavenger in the body that improves immunity and reduces the total amount of inflammatory substances in the body. GSH is an important factor in measuring the size of the body’s antioxidant capacity. It is an important antioxidant and free radical scavenger that improves immunity and reduces the total content of inflammatory substances. GSH depletion is the main cause of ferroptosis. Combined with the reduced content of Fe^2+^ induced by XYS, we hypothesised that XYS reduced cellular ferroptosis by improving the body’s ability to scavenge oxygen free radicals.

Grsf1 regulates mitochondrial gene expression at the post-transcriptional level and plays a role in cellular senescence by regulating mitochondrial homeostasis, the reduction of which can lead to abnormal mitochondrial structure and function (Antonicka et al., 2013; Noh et al., 2018). GPX4 is associated with gene regulation and antioxidant defence. XYS increased GPX4 levels in liver tissues, but this effect was blocked when Grsf1 was disrupted in the present study. This result suggests that Grsf1 positively correlated with GPX4 expression, which is similar to previous findings that the promotion of GPX4 by XYS also occurred via Grsf1 (Yin et al., 2019). The effect of XYS on mitochondrial synthesis and the expression of ferroptosis-related proteins was abolished in the absence of Grsf1.

However, there are some limitations in this study. There have been an increasing number of reports about the potential hepatotoxicity of TCM botanical drugs (Wu et al., 2019), but the results of a valid causality assessment are rarely provided (Frenzel and Teschke, 2016). Regulation of TCM botanical drugs should be strengthened, and RUCAM should be performed in suspected cases of liver injury (Frenzel and Teschke, 2016). RUCAM represents a structured, standardised, quantitative, and liver injury specific diagnostic tool that provides a final score and final causality rating by assessing seven key factor characteristics of liver injury (Danan and Teschke, 2016). RUCAM was used to determine causality in 81,856 DILI and 14,029 botanical drug-induced liver injury cases worldwide between 1993 and mid-2020, and the 2016 updated RUCAM should be used as the gold standard for the diagnosis of DILI (Shahbaz et al., 2017; Teschke and Danan, 2021). For example, Zhang used the updated RUCAM to analyse causality in TCM botanical drug-induced liver injury cases, of which 26 patients were highly probable, and 28 patients were probable (Zhang et al., 2016). A case-control study used an updated RUCAM to assess causality in botanical drug-induced liver injury cases of *Polyflorum multiflorum* (Jing et al., 2019). Future studies based on the updated RUCAM must be performed to confirm whether XYS causes liver injury. Our study only provided some evidence that XYS had hepatoprotective effects, and the Grsf1 pathway is only one of the pathways that affect hepatoprotection. XYS may be a prescription that interferes with ATDH. Future research should focus on the following questions. 1) Does XYS play a role in other novel hepatoprotective pathways? 2) Studies with high-quality prospective designs should be performed based on the updated RUCAM, and the use of XYS should be closely monitored to balance risks and benefits. 3) High-quality, large-sample RCT studies are required to evaluate the effectiveness and safety of XYS intervention in ATDH.

## Conclusion

Our findings suggested that XYS reduced liver injury in AT drug-induced liver-injured mice by increasing Grsf1 and promoting balanced regulation of intracellular ROS levels *via* GPX4, which reduced oxidative stress.

## Data Availability

The original contributions presented in the study are included in the article/Supplementary Material, further inquiries can be directed to the corresponding authors.

## Glossary

AC: anti-tuberculous chemotherapy
ALT: alanine aminotransferase
ANOVA: analysis of variance
ASP: Angelica sinensis polysaccharides
AST: aspartate aminotransferase
AT: anti-tuberculosis
ATDH: anti-tuberculosis drug-induced hepatotoxicity
DCF: 2′,7′-dichlorofluorescein
DCFH-DA: dichloro-dihydro-fluorescein diacetate
DILI: drug-induced liver injury
FTH1: ferritin heavy chain 1
GPX4: glutathione peroxidase 4
Grsf1: G-rich RNA sequence binding factor 1
GSH: glutathione
HE: haematoxylin and eosin staining
HPLC-DAD: high-performance liquid chromatography with diode array detection
HRP: horseradish peroxidase
NRF1: nuclear respiratory factor 1
PCG-1α: peroxisome proliferator-activated receptor γ coactivator 1 alpha
RCT: randomised controlled trial
ROS: reactive oxygen species
RUCAM: Roussel Uclaf Causality Assessment Method
SD: standard deviation
SOD: superoxide dismutase
TB: tuberculosis
TCM: traditional Chinese medicine
TFAM: mitochondrial transcription factor A
TfR1: transferrin receptor
MDA: malondialdehyde
WHO: World Health Organization
XYS: Xiao-Yao-San
