# Supplementary figures and images for: Xiao-Yao-San protects against anti-tuberculosis drug-induced liver injury by regulating Grsf1 in the mitochondrial oxidative stress pathway
Source: Front Pharmacol. 2022 Sep 1;13:948128. doi: 10.3389/fphar.2022.948128 (PMC9475289; doi:10.3389/fphar.2022.948128)

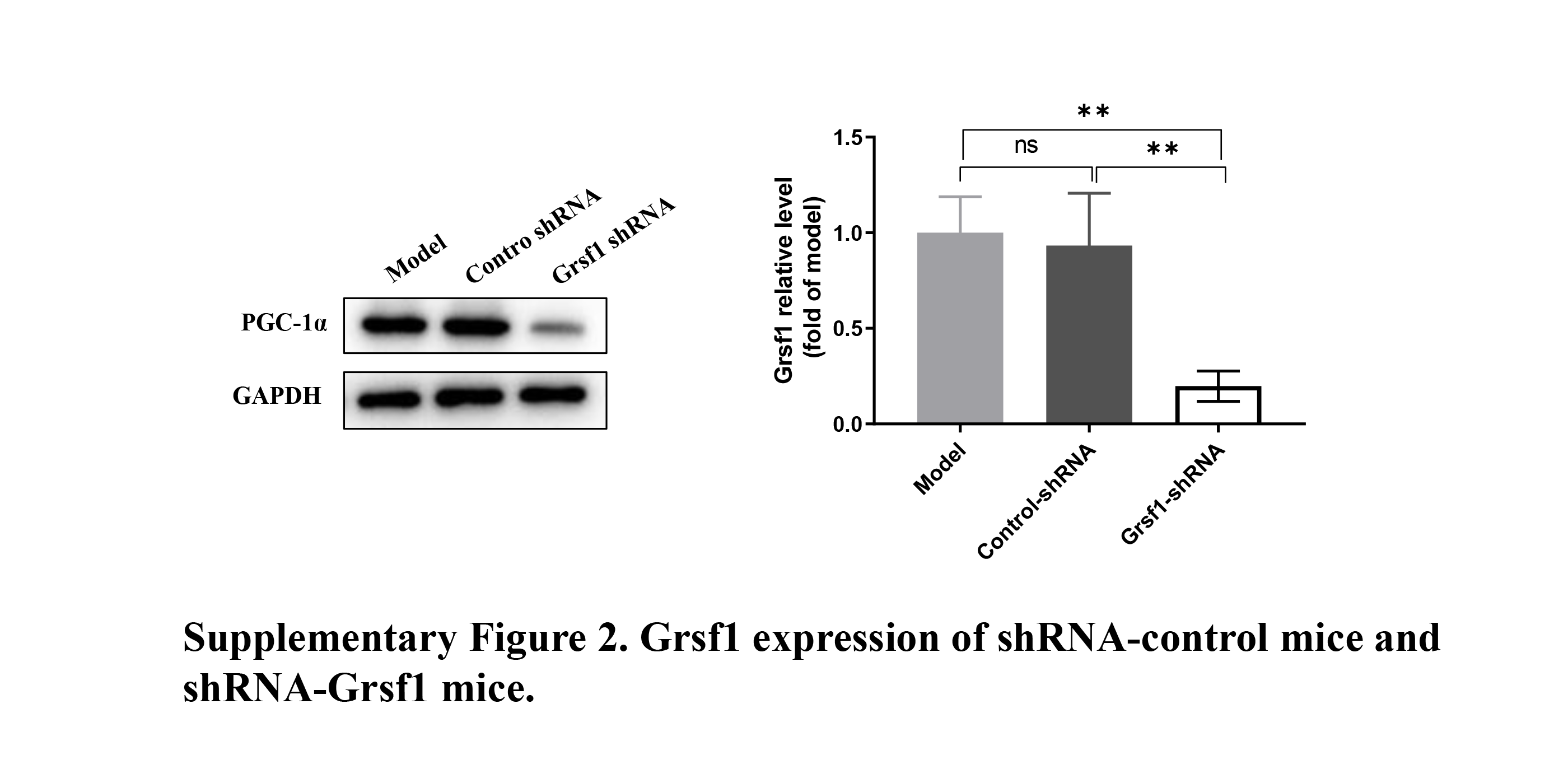

Supplement: Supplementary file 1 [file Image2.TIF]

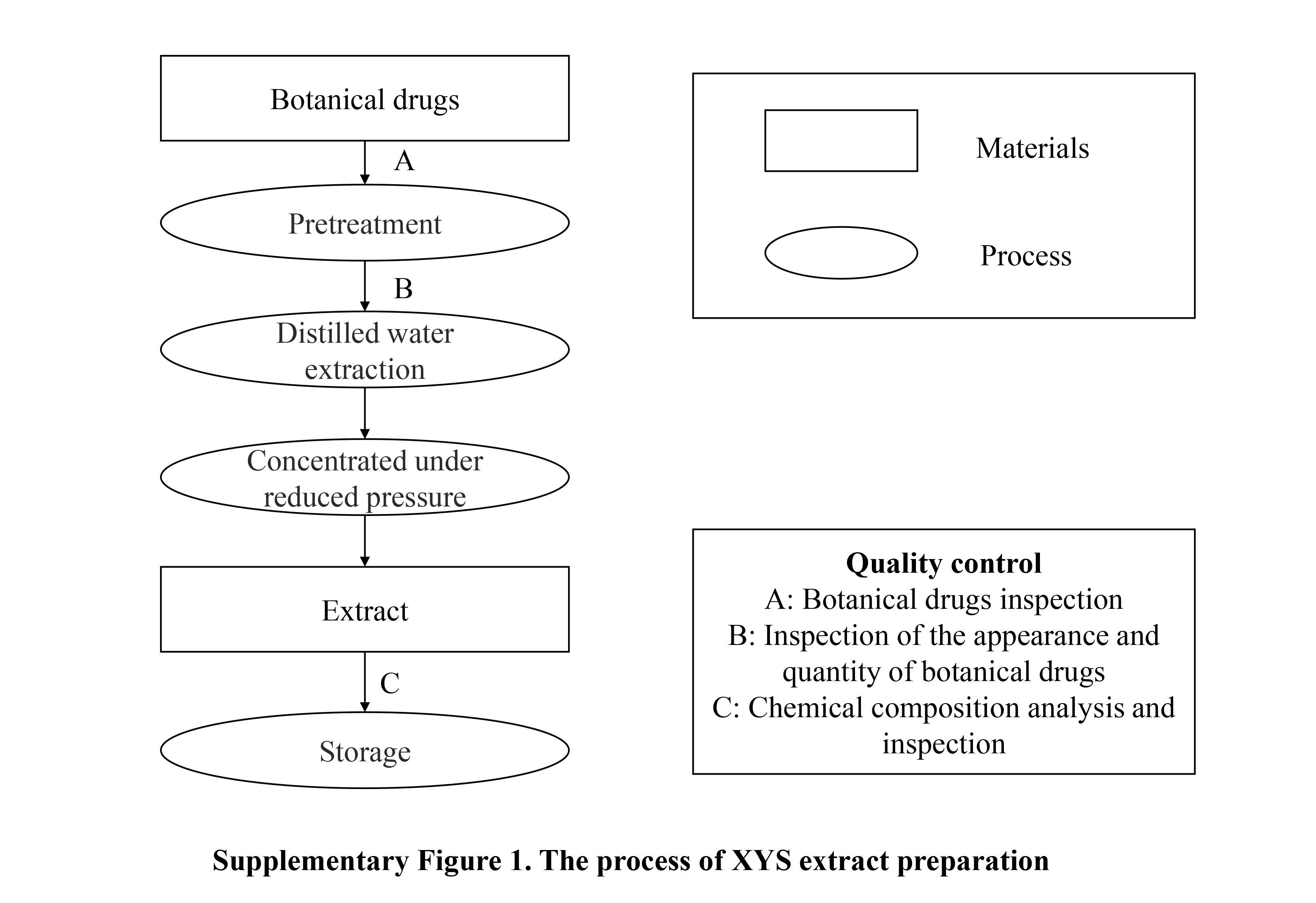

Supplement: Supplementary file 2 [file Image1.TIF]
